# Integrated Volatile Metabolomics and Bacterial Community Profiling Identify Potential Quality Markers for Geographical Discrimination of *Microcos paniculata* L. Herbal Tea

**DOI:** 10.3390/foods15203744

**Published:** 2026-10-21

**Authors:** Yihang He, Pu Xu, Jianing Zhou, Jiayi Zheng, Riyan Yuchi, Xintong Wang, Binbin Wei, Song Cang

**Affiliations:** 1 School of Pharmacy, China Medical University, No. 77 Puhe Road, Shenyang 110122, China; heyihang0302@outlook.com (Y.H.); 18635962182@163.com (P.X.); linda.zhou2005@outlook.com (J.Z.); zhengjiayikin@icloud.com (J.Z.); ryuchi01@outlook.com (R.Y.); wangxintong050321@163.com (X.W.); 2 Department of Sanitary Inspection, School of Public Health, Shenyang Medical College, No. 146 North Huanghe Street, Shenyang 110034, China

**Keywords:** *Microcos paniculata* L., volatile metabolites, bacterial community, antioxidant activity, origin traceability

## Abstract

*Microcos paniculata* L. (ML) is an edible herbal tea plant with regional differences in flavor and bioactive quality. However, the chemical and microbial factors associated with these differences remain unclear. This study integrated HS-GC-Orbitrap-MS/MS, 16S rRNA sequencing, sensory evaluation, color analysis, and antioxidant assays to characterize ML samples from Guangdong, Guangxi, and Yunnan, China. Guangdong samples were characterized by the highest total phenolic and flavonoid contents and superior antioxidant capacity, whereas Yunnan samples showed the best sensory performance, particularly for aroma and taste attributes. A total of 133 volatile constituents were tentatively identified, mainly comprising olefins and cycloolefins, aromatic compounds, terpenes, alcohols, and ketones. Chemometric analysis revealed clear origin-dependent separation and identified 12 differential volatile compounds, including p-cymene, L-limonene, α-phellandrene, dimethyl sulfide, and ethylbenzene. Bacterial community analysis showed distinct origin-associated patterns, and several bacterial taxa were correlated with characteristic volatile compounds and quality traits. Functional prediction and multivariate correlation analysis suggested potential associations among environmental factors, bacterial communities, volatile metabolites, and antioxidant properties. These findings provide a foodomics-based framework for understanding geographical quality variation in ML and offer potential markers for origin traceability and quality evaluation of edible herbal tea resources.

## 1. Introduction

*Microcos paniculata* L. (ML) is a traditional edible plant widely consumed as an herbal tea in the Lingnan region of China. ML was approved for use as a food and beverage ingredient by the National Health Commission of the People’s Republic of China in 2010 (National Health Commission Policy Document No. 3 of 2010). The dried leaves are rich in bioactive compounds such as flavonoids, phenolic acids, and terpenoids [1]. ML exhibits various beneficial effects, including antioxidant, anti-inflammatory, and lipid-lowering activities [2,3]. According to the theory of geo-authentic (“Daodi”) medicinal materials, geographical origin critically influences plant chemical composition and quality. However, the associations underlying regional differences in the flavor and functional quality of ML remain insufficiently studied. In particular, the role of epiphytic microbial communities in these differences is still unclear.

The plant leaf surface harbors a highly complex and active epiphytic microbial community [4]. These microorganisms interact with plant exudates and environmental conditions and may be associated with host secondary metabolism. Recent studies on tea and fruit have reported associations between epiphytic microorganisms and volatile profiles [5,6]. However, direct microbial contributions to volatile formation require experimental validation. For ML, integrated evidence linking geographical origin, epiphytic microbiota, and quality remains limited.

We hypothesized that *Microcos paniculata* L. from different geographical origins would show distinct epiphytic bacterial communities and volatile profiles. This study integrated HS-GC-Orbitrap-MS/MS, 16S rRNA sequencing, electronic-nose and sensory analyses, color measurement, and antioxidant assays to compare samples from Guangdong (GD), Guangxi (GX), and Yunnan (YN). Redundancy analysis and correlation networks were used to explore associations among regional descriptors, bacterial communities, metabolites, and quality traits. PICRUSt2 and FAPROTAX predictions were treated as hypothesis-generating rather than direct measurements of microbial activity.

## 2. Materials and Methods

### 2.1. Materials

All chemicals and solvents used in this study were of analytical grade and were used without further purification. Sodium carbonate (Na_2_CO_3_, 497-19-8), sodium nitrite (NaNO_2_, 7632-00-0), aluminium nitrate (AlNO_3_, 13473-90-0), ethanol (C_2_H_6_O, 64-17-5), hydrochloric acid (HCl, 7647-01-0), ferric chloride hexahydrate (FeCl_3_·6H_2_O, 10025-77-1), rutin (C_27_H_30_O_16_, 153-18-4), Folin–Ciocalteu (C_6_H_6_O, 12111-13-6), gallic acid (C_7_H_6_O_5_, 149-91-7), sodium hydroxide (NaOH, 1310-73-2), potassium persulfate (K_2_O_8_S_2_, 7727-21-1), 6-Hydroxy-2,5,7,8-tetramethylchroman-2-carboxylic acid (Trolox), acetic acid buffer were supplied by Yuanye Biotechnology Ltd. (Shanghai, China). The amount of organic solvent was kept consistent among samples to minimize its influence on antioxidant measurements.

### 2.2. Sample Preparation and Extraction

The ML samples were acquired from six distinct batches across three Chinese provinces: Guangdong (GD), Guangxi (GX), and Yunnan (YN). All six batches were collected within the same autumn harvesting period at their respective local sampling sites. The geographical and ecological characteristics of the three production regions are summarized in Table 1. A temperature of 4 °C was maintained for all samples until formal experimentation and processing. The ML samples were manually sorted to remove stems, and only leaf lamina tissues were retained. A uniform powder was achieved by pulverizing the selected leaves with a grinder and passing them through an 80-mesh sieve. For the extraction, 0.15 g of the resulting powder was then added to 5 mL of 70% ethanol. Ultrasonic-assisted extraction was conducted for 30 min under optimized parameters (500 W, 40 kHz). Post-extraction, the mixtures were centrifuged at 12,000 *g* for 30 min at 4 °C [7]. The supernatants were collected as ML extracts and stored sealed at 4 °C to maintain stability until analysis.

### 2.3. TPC Detection

Total phenolic content (TPC) was determined with slight methodological modifications [8]. TPC was determined using Folin–Ciocalteu reagent. Diluted extract (1 mL) was mixed with diluted reagent (2 mL) and sodium carbonate (2 mL, 75 g/L), vortexed, and incubated for 40 min in the dark. Absorbance was measured at 760 nm, and results were expressed as gallic acid equivalents (GAE).

### 2.4. TFC Detection

TFC was determined based on the method of [9] with minor modifications. Briefly, 0.8 mL of diluted sample extract was mixed with 0.3 mL of 5% sodium nitrite and incubated for 6 min in the dark. Then, 0.3 mL of 10% aluminum nitrate and 4 mL of 1 M sodium hydroxide were added. The volume was adjusted to 10 mL with 60% ethanol, incubated for 10 min, and measured at 510 nm. TFC was expressed as rutin equivalents.

### 2.5. DPPH Radical Scavenging Activity

The DPPH radical-scavenging activity was evaluated based on the procedure established by [10], employing minor modifications. Briefly, 0.5 mL of the suitably diluted ML sample solution was combined with 2.5 mL of a freshly prepared 80 μM DPPH ethanol solution. The resultant mixture was incubated in the dark for 2 h. Absorbance was subsequently measured at 515 nm using a microplate reader. The scavenging capacity was quantified using a Trolox standard calibration curve and expressed as micromolar Trolox equivalents per gram (μmol TE/g).

### 2.6. ABTS Radical Scavenging Activity

The ABTS radical-scavenging activity was evaluated based on the procedure established by [11], employing minor modifications. The ABTS working solution was prepared by mixing equal volumes of 7 mmol/L ABTS and 2.45 mmol/L potassium persulfate solutions. After dilution, the mixed solution was kept in the dark at ambient temperature for 12 h to reach a plateaued absorbance of 0.70 ± 0.02 at 734 nm. To perform the assay, 200 μL of each diluted sample was combined with 2.8 mL of the prepared ABTS solution. Following a 6 min reaction at 25 °C under dark conditions, the absorbance value was recorded at 734 nm. Antioxidant capacity was calculated against the Trolox calibration curve and presented as micromolar Trolox equivalents per gram (μmol TE/g) [12].

### 2.7. FRAP Detection

The ferric reducing antioxidant power (FRAP) assay was performed based on the method of [13] with minor modifications. FRAP activity was measured using a TPTZ-based assay. The FRAP working solution was prepared by mixing TPTZ solution, FeCl_3_·6H_2_O, and acetate buffer at 1:1:10 and preheated at 37 °C. Diluted sample (200 μL) was added to 2.8 mL of working solution, incubated for 30 min, and measured at 593 nm. Results were expressed as μmol TE/g.

### 2.8. Color Difference Analysis

Quantitative analysis of color parameters (L*, a*, b*) was performed using a calibrated YS6010 color difference meter (Guangdong 3NH Technology Co., Ltd., Guangzhou, China) under D65 light source and 10° viewing angle conditions. The instrument was calibrated with a certified white reference tile before each measurement. ML infusions from different geographical origins were transferred to a 10 mm optical path quartz cuvette, with three replicate measurements per sample. Random sample measurement was adopted to minimize systematic errors. ΔL, Δa, and Δb correspond to differences in L*, a*, and b* values between the two samples, respectively. Total color difference is expressed as ΔE, calculated using the following formula:

ΔE = √(ΔL^2^ + Δa^2^ + Δb^2^) (1)

L*: Lightness (0 = black, 100 = white)

a*: Red–green chromaticity (+a = red, −a = green)

b*: Yellow–blue chromaticity (+b = yellow, −b = blue)

When ΔE < 1, the color difference was generally considered imperceptible. When 1 ≤ ΔE ≤ 2, the difference was slight and may be perceived by professional observers. When 2 < ΔE ≤ 3.5, the difference was noticeable and could be perceived by ordinary people under comparison. When ΔE > 3.5, the difference was significant [14].

### 2.9. Electronic Nose Analysis

A PEN3 portable electronic nose system (Airsense, Schwerin, Germany) analyzed the volatile component fingerprints. Detailed sensor information is presented in Table S3. The system features 10 metal-oxide sensors (W1C, W5S, W3C, W6S, W5C, W1S, W1W, W2S, W2W, W3S) responsive to different volatile classes. Detection parameters included: 5 s sample preparation, 120 s data acquisition, 60 s sensor cleaning, and a 400 mL/min carrier gas flow [10]. A randomized block design was used with six biological replicates per sample (2 g each) under 25 ± 1 °C and 50 ± 5% relative humidity. Steady-state sensor responses were extracted as features; reciprocals were used for W1C, W3C, and W5C, while other sensors were used directly. PCA was applied for pattern recognition, and differences were analyzed by one-way ANOVA with Tukey’s HSD test (*p* < 0.05).

### 2.10. Sensory Evaluation

As ML is consumed as tea, sensory evaluation followed GB/T 23776-2018 for powdered tea and was conducted by six trained panelists (3 males, 3 females; 22–25 years). This training aimed to enhance their ability to discriminate and describe quality attributes, including appearance, infusion color, aroma, taste, and infused leaves [15]. The national standard specifies the evaluation procedures, protocols, and criteria for sensory attributes of powdered tea. For the evaluation process, 0.6 g of ML sample was weighed into a 240 mL tea tasting bowl. Then, 150 mL of boiling water was poured from a 150 mL tea tasting cup. The infusion time was set at 3 min. Subsequently, panelists evaluated the infusion color, aroma, and taste of ML in sequence.

The sensory evaluation was conducted in accordance with the ethical principles of the World Medical Association’s Declaration of Helsinki, and institutional approval was not required. Participants were fully informed of the study procedures and potential risks, took part voluntarily, and provided written or oral informed consent. Data confidentiality was maintained, and participants could withdraw at any time without penalty. All ML samples were food-grade and suitable for consumption. In addition, the sensory evaluation in this study was performed by six trained panelists. It should be noted that the panel size is relatively limited, which constitutes one limitation of the present study. Further sensory tests with a larger panel cohort would be helpful to validate these sensory-related observations.

### 2.11. Analysis of Volatile Metabolites (HS-GC-Orbitrap-MS/MS)

2g of 80-mesh ML powder were placed in a 20 mL headspace vial with a magnetic stir bar, and heated at 60 °C for 5 min. 1ml of headspace gas was sampled by the auto-sampler for analysis. A TG-5MS capillary column was used. High-purity helium was used as the carrier gas at a constant flow rate of 1.0 mL/min. The injector temperature was maintained at 200 °C. The oven temperature was initially held at 35 °C for 2 min, increased to 100 °C at 2 °C/min, then raised to 240 °C at 5 °C/min and held for 2 min. Mass spectrometry conditions: electron impact (EI) ion source at 230 °C, mass scanning range of *m*/*z* 30–600 [16]. 2.0 g of ML powder was placed in a 20 mL headspace vial and equilibrated at 80 °C for 30 min. Volatile compounds were tentatively identified by mass spectral matching against the NIST 17 library, using a similarity score of ≥80% as the primary annotation criterion. An n-alkane homologous series was analyzed under the same chromatographic conditions during the original analysis, and experimental retention indices were calculated and compared with available reference/library RI information as an additional criterion supporting compound annotation. However, the complete compound-specific experimental RI dataset and the corresponding original n-alkane retention-time records could not be retrieved for inclusion in the present manuscript. Therefore, individual experimental RI values and ΔRI values are not reported. Available database/literature RI values are provided in Table S3 as reference information. Because authentic reference standards were not analyzed, all volatile-compound assignments are considered tentative.

### 2.12. Microbial Community Analysis

Epiphytic microorganisms were detached from ML leaves using sterile phosphate-buffered saline, followed by filtration, centrifugation, and DNA extraction. The bacterial 16S rRNA V3–V4 regions were amplified with primers 338F/806R, purified, and subjected to paired-end sequencing on the Illumina NovaSeq 6000 platform (Illumina, Inc., San Diego, CA, USA).

Bioinformatic analysis: Raw sequencing data were processed using QIIME2 [17]. Amplicon sequence variants (ASVs) were generated via the DADA2 method, and taxonomic annotation of ASVs was conducted against the SILVA database. α-diversity and β-diversity analyses were completed in QIIME2. LEfSe analysis was applied to identify differentially abundant microbial genera among groups (LDA > 2.0, *p* < 0.05). PICRUSt2 and FAPROTAX tools were used to predict microbial community functional profiles, followed by KEGG pathway enrichment analysis.

### 2.13. Data Analysis

Multivariate analysis of HS-GC-Orbitrap-MS/MS data was performed using SIMCA 14.1. PCA was used for unsupervised visualization, and pairwise OPLS-DA models were constructed for GD, GX, and YN. Candidate differential compounds were screened using VIP ≥ 1, fold change ≥ 2 or ≤ 0.5, and *p* < 0.05. Each biological batch was analyzed in triplicate, and technical replicates were averaged before between-origin comparisons. One-way ANOVA followed by Tukey’s HSD test was used where applicable. HCA heatmaps were generated with TBtools v2.1; graphs were prepared using Origin 2018 and GraphPad Prism 10.4; RDA was performed in R; and correlation networks were constructed in Cytoscape 3.9.1.

## 3. Results and Discussion

### 3.1. Total Phenolic and Flavonoid Contents Analysis

**Figure 1:**
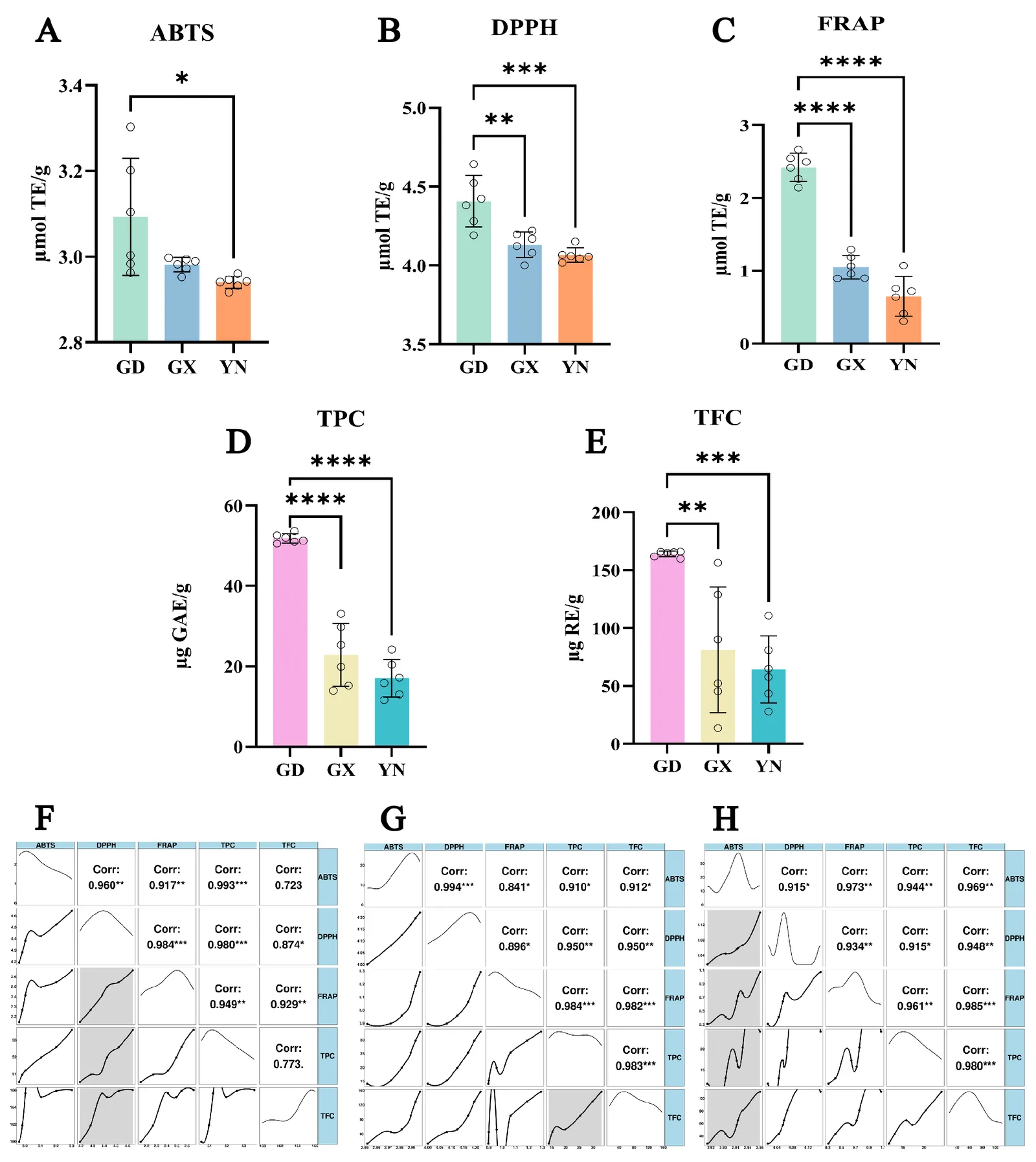
The antioxidant (ABTS, DPPH, FRAP) results of ML extract are shown in graphs (**A**–**C**); Graphs of TPC and TFC results of ML extract (**D**,**E**); Pearson correlation analysis of antioxidant activities across GD, GX, and YN groups (**F**–**H**) (ns, No significant correlation; * Correlation is significant at the *p* < 0.05; ** Correlation is significant at the *p* < 0.01; *** Correlation is significant at the *p* < 0.001; **** Correlation is significant at the *p* < 0.0001, *n* = 6).

As shown in Figure 1, physicochemical analysis revealed origin-associated differences in the accumulation of secondary metabolites in ML samples. Detailed specific values are shown in Table S1. The GD samples exhibited an significantly higher levels in TPC and TFC (*p* < 0.05). Specifically, the TPC of the GD group reached 51.25 ± 0.33 μg GAE/g, which was 2.3 and 2.8 times higher than that of the GX (21.83 ± 6.74 μg GAE/g) and YN groups (18.18 ± 4.77 μg GAE/g), respectively (Figure 1D). The TFC of the GD group (164.13 ± 1.32 μg RE/g) was also significantly higher than the GX (76.93 ± 45.74 μg RE/g) and YN groups (69.01 ± 26.36 μg RE/g) (Figure 1E).

### 3.2. Antioxidant Capacity and Correlation Analysis

The higher TPC and TFC in GD samples was accompanied by stronger in vitro antioxidant capacity. Detailed specific values are shown in Table S1. The mean ABTS, DPPH, and FRAP values in GD were 3.09 ± 0.14, 4.41 ± 0.16, and 2.42 ± 0.19 μmol TE/g, respectively (Figure 1A–C). GD was higher than YN in ABTS and higher than both GX and YN in DPPH and FRAP.

Correlation analysis further clarified the contribution of phenolics and flavonoids to ML’s antioxidant activity (Figure 1F–H). In GD samples, TPC and TFC were strongly correlated with ABTS, DPPH, and FRAP activities, indicating their major contribution to antioxidant capacity. Although similar positive correlations were observed in other samples, the lower coefficients suggest that antioxidant-related compounds in ML may vary by geographical origin, highlighting the complexity of its quality formation [18].

### 3.3. Color Difference Evaluation

**Figure 2:**
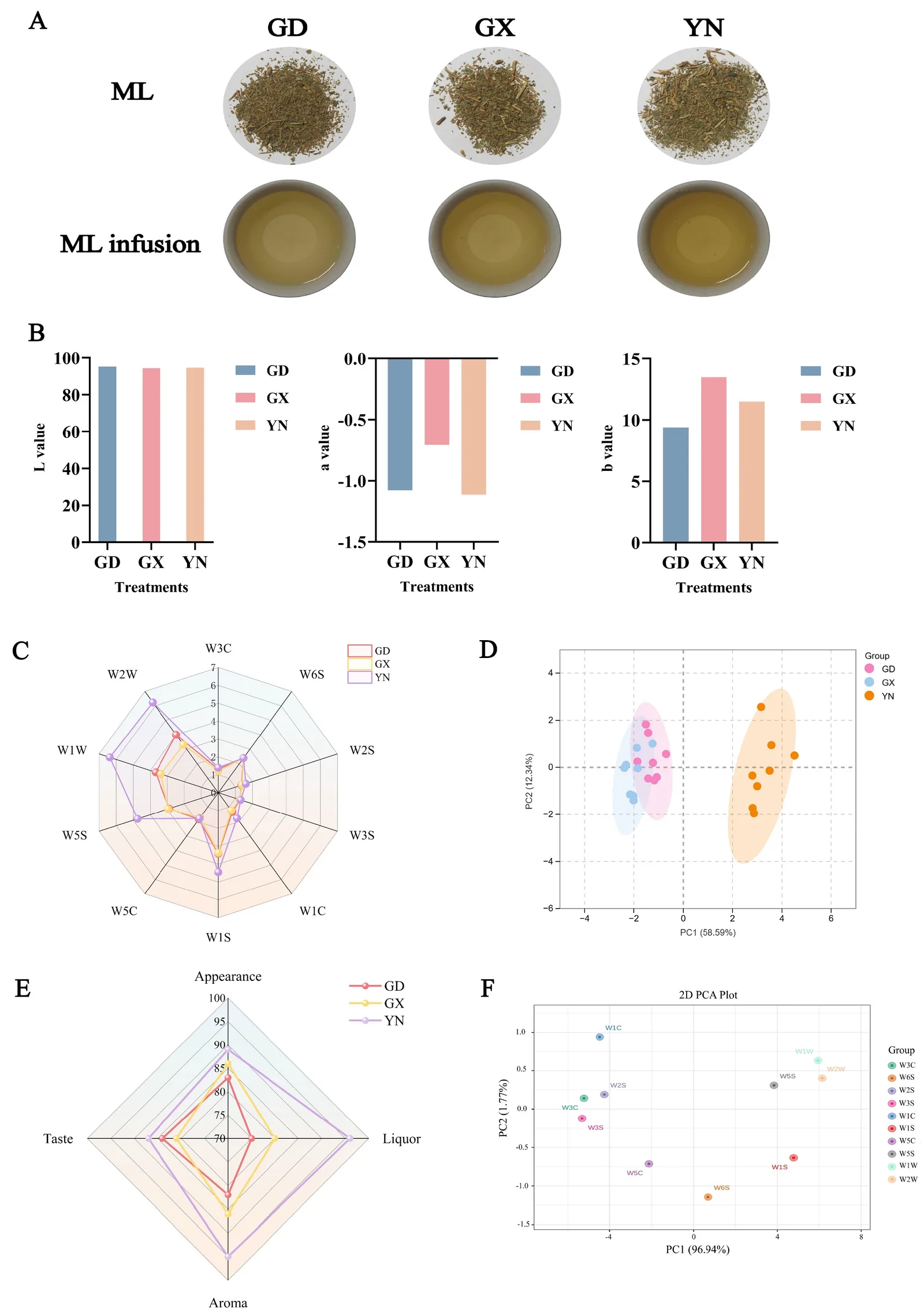
Visual and sensory evaluation of ML samples and their infusions from different producing areas. Appearance of raw ML materials and prepared infusions (**A**); Color parameters (L*, a*, b*) of ML infusions (**B**); radar plot of electronic nose responses to ML infusions (**C**); PCA score plot of the electronic nose data (**D**); radar plot of sensory evaluation of ML infusions (**E**); loading plot of the electronic nose sensors (**F**).

Infusion appearance and CIELAB coordinates differed among origins (Figure 2A,B). The GX samples recorded the lowest L* value (87.75 ± 0.44) and the highest b* value (13.91 ± 0.43). The three groups had similar L* values, whereas GX showed the highest b* value, YN was intermediate, and GD showed the lowest b* value. The a* values were negative for all groups, indicating a green component.

Further comparative analysis of the colour differences between the ML infusions of the two regions showed that the ΔE values between GD and GX, GD and YN, and GX and YN were 4.18, 2.13, and 2.04, respectively (Table S2).

### 3.4. Electronic Nose Evaluation

The electronic nose records a cross-sensitive response pattern from a metal-oxide sensor array and is therefore suitable for sample-level odor discrimination rather than compound-specific identification [19,20].

PCA of the electronic-nose responses separated the three origin groups (Figure 2D). The cumulative contribution of PC1 and PC2 exceeded 85%, indicating that geographical origin contributed to differences in the overall odor profiles. Loading analysis revealed that the W5S (nitrogen oxides) and W2W (aromatics/organic sulfides) sensors contributed most to regional discrimination (Figure 2F). The GD samples showed significantly higher responses on the W5S sensor (Figure 2C). This indicates that nitrogen oxides or related secondary metabolites contribute to its distinct olfactory profile. Pairwise OPLS-DA models further visualized differences in electronic-nose fingerprints (Figure S1A–C). In the 200-permutation tests, permuted Q^2^ values were lower than the original values (Figure S1D–F), indicating no obvious overfitting within the present dataset.

### 3.5. Sensory Evaluation Characterization

To further validate the flavor attributes reflected by the physicochemical analysis and electronic nose results, a trained sensory panel performed multidimensional scoring of the samples. The YN samples showed the best overall sensory performance, achieving the highest total score (84.17 ± 1.17), with significantly higher aroma (25.17 ± 0.41) and taste scores (42.00 ± 0.89) than the other two groups (Figure 2E).

GD obtained a total sensory score of 81.33 ± 1.63, whereas GX had the lowest total score (80.50 ± 1.38). These results indicate origin-associated sensory differences, with YN showing the most favorable combined aroma and taste profile. Overall, YN showed the strongest sensory performance, whereas GD was characterized by higher antioxidant-related indices. These complementary differences motivated the subsequent volatile and bacterial community analyses.

### 3.6. Volatile Metabolite Profiling Analysis

**Figure 3:**
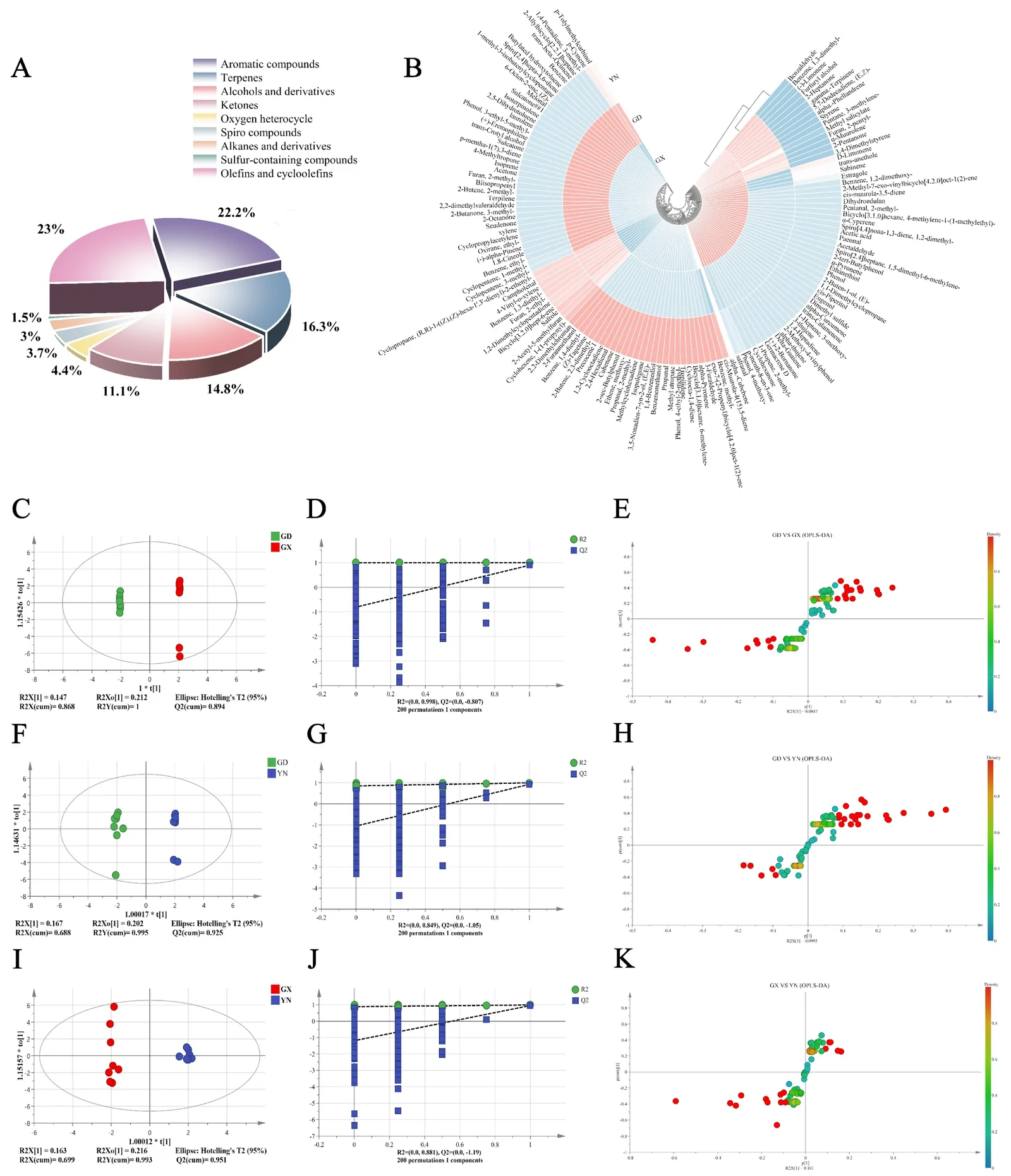
Volatile metabolite profiling and chemometric analysis of ML samples. Chemical classification of volatile compounds (**A**); clustered heatmap of 133 volatile compounds (**B**); OPLS-DA score plots, permutation tests, and S-plots for GD vs. GX (**C**–**E**), GD vs. YN (**F**–**H**), and GX vs. YN (**I**–**K**). Red dots in the S-plots represent variables with VIP > 1. In the OPLS-DA score plots, the asterisk (*) in the axis labels denotes multiplication by the scaling coefficient displayed in SIMCA and does not indicate statistical significance. The terms t[1] and to[1] represent the first predictive and first orthogonal component scores, respectively.

Volatile metabolites of regional ML samples were analyzed via HS-GC-Orbitrap-MS/MS. A total of 133 volatile compounds were tentatively identified through comparative spectral matching. GD contained 71 annotated compounds, GX 68, and YN 53. The compounds were assigned to nine classes, including olefins and cycloolefins, aromatic compounds, terpenes, alcohols and derivatives, and ketone (Table S4, Figure 3A). The heatmap showed origin-associated abundance patterns (Figure 3B). It clearly visualized the distribution patterns of volatile compounds across the three geographical origins, indicating that geographical origin was associated with distinct volatile metabolic fingerprints of Microcos paniculata leaves [21].

To further identify the key metabolic variables responsible for origin-dependent differences, an OPLS-DA model was constructed. The score plot showed clear spatial separation among samples from the three regions, with PC1 explaining 42.6% of the variance and effectively separating the GD group from the GX and YN groups, whereas PC2 explained 18.3% of the variance and distinguished the GX group from the YN group. All three pairwise OPLS-DA models showed clear separation between groups and satisfactory predictive performance (Figure 3C,F,I). A subsequent 200-time permutation test showed no evidence of overfitting, further confirming the reliability of the multivariate statistical analysis (Figure 3D,G,J).

### 3.7. Key Differential Markers for Origin Discrimination

To screen differential compounds between pairwise regions, the OPLS-DA model in SIMCA-P 14.1 software was used to calculate VIP values. Tentatively identified compounds with VIP ≥ 1 were initially selected as candidate differential volatile compounds, which are highlighted in red in the S-plot (Figure 3E,H,K) (Figure S2A).

The GD samples were characterized by higher relative abundances of p-cymene and L-limonene (Figure S2B). Terpenoids generally contribute fresh citrus-like and herbal notes to plants, which was consistent with the high response of the W5S sensor in the electronic nose analysis and the fresh aroma perceived in the sensory evaluation of the GD samples [22]. Other terpene-related compounds are also associated with the volatile characteristics of GD samples.

For the YN group, ethylbenzene and dimethyl sulfide showed relatively high accumulation in the YN sample (Figure S2C). These compounds are often linked to non-enzymatic reactions during drying and processing and are associated with the distinctive flavor depth and aroma persistence of Yunnan samples [23]. The GX group exhibited a relatively balanced but lower-abundance metabolic profile, without a clearly dominant marker compound (Figure S2D).

### 3.8. Microbial Community Characteristics and Biomarkers

#### 3.8.1. Geographical Variation in Epiphytic Microbial Diversity

**Figure 4:**
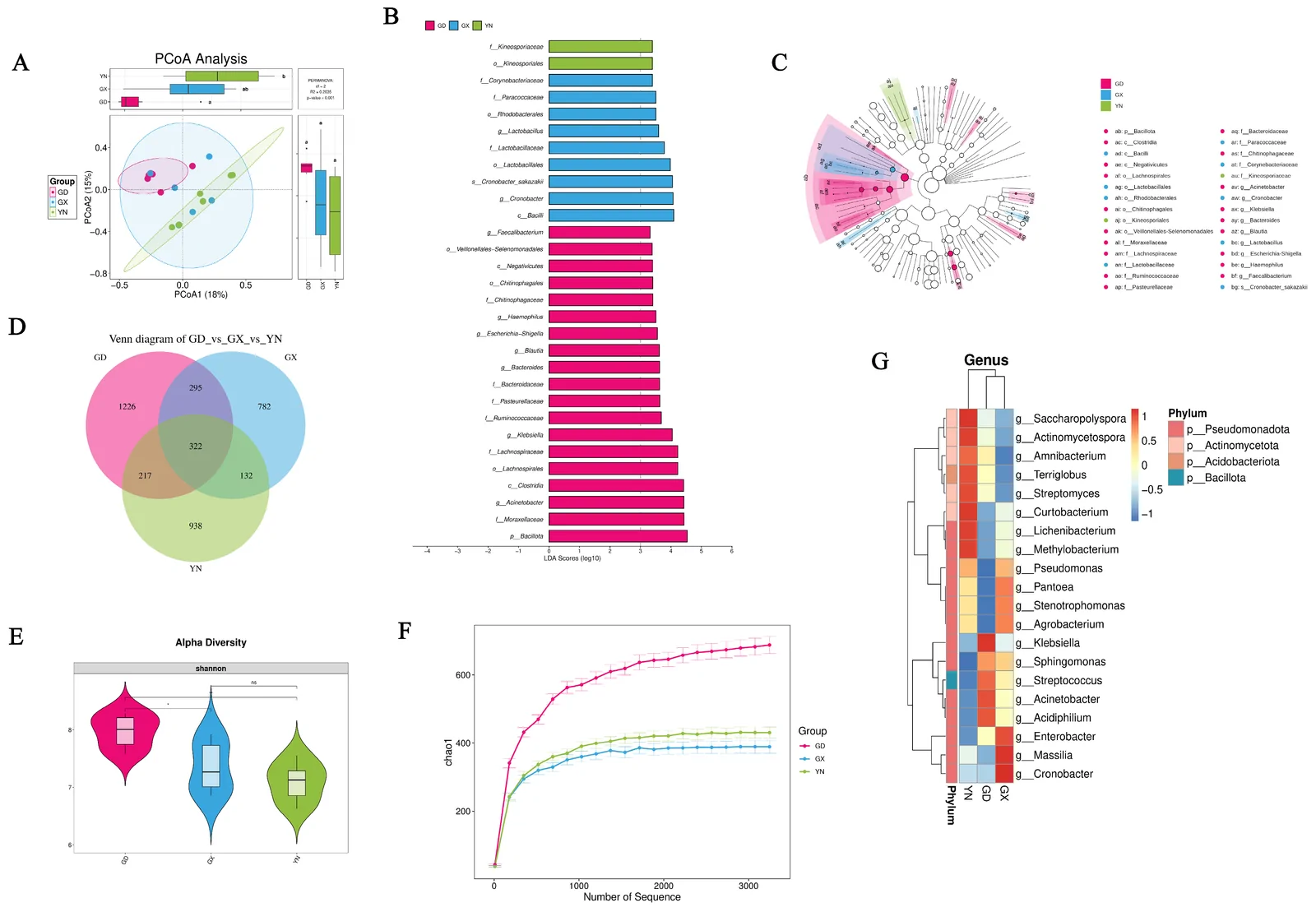
Microbial community characteristics of ML from different geographical origins: PCoA of microbial communities based on Bray–Curtis distance (**A**); rarefaction curves of microbial communities (**B**); Circos plot of microbial community taxonomic hierarchy (**C**); Venn diagram of microbial OTUs (**D**); violin plots of Shannon and Chao1 indices of microbial communities (**E**); clustered heatmap of microbial community relative abundances at the genus level (ns, No significant correlation; * Correlation is significant at the *p* < 0.05, *n* = 6) (**F**); LDA score plot of LEfSe analysis (**G**).

The alpha diversity of epiphytic bacterial communities on the surface of ML was evaluated by 16S rRNA gene sequencing. The YN samples showed significantly higher Shannon (4.48 ± 0.21) and Chao1 indices (348.12 ± 15.60) than the GD and GX samples (*p* < 0.05, Figure 4E). These results suggest that environmental conditions in YN are associated with the maintenance of a highly complex epiphytic microbial ecosystem. In contrast, the GD samples had a relatively low Shannon index of 3.52 ± 0.18.

This reduction in diversity is associated with strong selective pressure. On the one hand, the hot and humid climate in Guangdong is associated with specific heat- and moisture-tolerant microorganisms. On the other hand, the highest levels of total polyphenols and total flavonoids in the GD samples are linked with habitat-filtering patterns of leaf-surface microorganisms, which could be linked to their broad-spectrum antimicrobial activity, concomitant with the dominance of specific microbial groups and lower abundance of sensitive taxa [24].

#### 3.8.2. Spatial Heterogeneity of Microbial Communities Based on PCoA

To further investigate similarities in community structure among different geographical origins, principal coordinate analysis (PCoA) based on Bray–Curtis distances was performed (Figure 4A). The first two principal coordinates, PC1 and PC2, explained 43.1% and 21.6% of the total community variation, respectively, accounting for 64.7% in total. In the PCoA score plot, samples from the three origins exhibited clear clustering patterns. The GD, GX, and YN groups occupied distinct regions of the plot, with replicate samples clustering closely within each group.

#### 3.8.3. Distribution Patterns of Dominant Genera

At the genus level, the microbial communities on the surface of ML exhibited strong origin-specific patterns, reflecting distinct metabolic microecologies among the different origins (Figure 4G). The GD group was characterized by the high relative abundance of Lactobacillus and Bacillaceae-related genera. As typical sugar-metabolizing bacteria, lactic acid bacteria are often associated with complex biochemical transformation processes [25]. The enrichment of these bacteria in the GD samples shows links to sugars and polysaccharides secreted by ML, alongside shifts in organic-acid levels, leaf-surface pH, and precursor transformation of volatile terpenes such as L-limonene. Meanwhile, the high abundance of Bacillaceae-related genera is correlated with their resistance mechanisms under high-temperature conditions [26].

The GX group showed a relatively simple community structure dominated mainly by proteobacterial genera such as Klebsiella and Escherichia-Shigella. This pattern bears relationships to the soil microbial background and the balance between dry and humid environmental conditions at the GX origin. The YN group exhibited the most diverse community structure, with enrichment of Bacteroidaceae, Lachnospiraceae, and Prevotella. Functional features related to complex plant polysaccharide degradation and carbon cycling correlate with these enriched taxa, which show correlations with the distinctive flavor depth observed in the sensory evaluation of the YN samples [27].

#### 3.8.4. Distribution Patterns and Taxonomic Biomarkers

To further elucidate the shared and unique microbial components, a Venn diagram was constructed (Figure 4D). A total of 322 core features were shared across all three regions. However, the GD group possessed a substantially higher number of unique features (1226) compared to GX (782) and YN (938), echoing the higher richness observed in the rarefaction analysis.

The taxonomic composition was further explored using a circular phylogenetic tree (Figure 4C) and a genus-level heatmap (Figure 4G). At the phylum level, Pseudomonadota and Bacillota were identified as dominant members across all groups [28]. Linear Discriminant Analysis (LDA) Effect Size (LEfSe) was employed to identify specific microbial biomarkers (LDA > 4.0) (Figure 4G). The YN and GX groups were characterized by an enrichment of Kineosporiaceae and taxa assigned to the genus Cronobacter, which are often associated with robust environmental adaptation and complex polysaccharide degradation. Some species within the genera Cronobacter and Actinomyces are recognized as opportunistic pathogens. However, the 16S rRNA amplicon sequencing used in this study does not provide sufficient resolution to identify these organisms at the species or strain level or to determine their viability and pathogenic potential. Therefore, the detection of these genera alone does not indicate the presence of pathogenic strains, and their potential safety implications require further investigation.

In contrast, the GD group showed a significant enrichment of taxa such as Negativicutes and Chitinophagales. The heatmap (Figure 4F) further highlighted the origin-specific enrichment of genera such as Saccharopolyspora and Actinomyces in the GD samples. These Actinomycetota-related genera are known for their ability to synthesize secondary metabolites and degrade recalcitrant organic matter [29]. In GD samples, these taxa were detected alongside higher polyphenol levels and a distinct terpenoid profile, suggesting a possible association between microbial community composition and the chemical characteristics of the leaves.

### 3.9. Microbial Function Prediction and Metabolic Pathway Analysis

**Figure 5:**
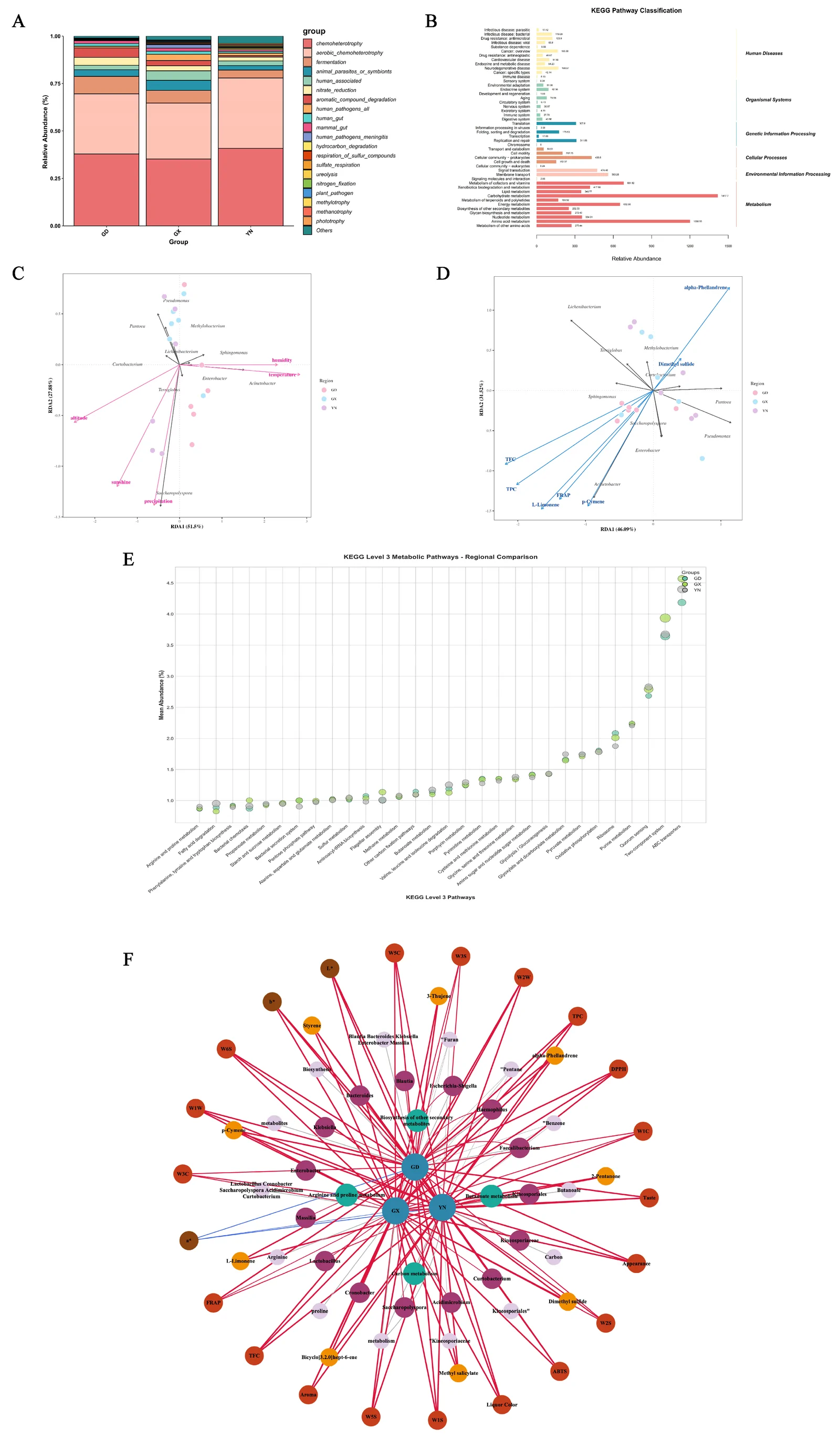
Microbial functional prediction and metabolic pathway analysis: Stacked bar plot of FAPROTAX-annotated relative functional abundances (**A**); bar plot of relative abundances of KEGG Level 2 pathway classifications (**B**); RDA linking environmental parameters with epiphytic microbial communities (**C**); RDA integrating differential microbial genera, quality indicators, and characteristic metabolites (**D**); bubble plot of enrichment in key KEGG Level 3 metabolic pathways (**E**); the integrated microbe-associated correlation network underlying quality formation in ML. Node colors represent different categories: blue (geographic origins), purple (signature microbial genera), green (KEGG metabolic pathways), orange (key flavor compounds), red (quality indices), brown (color parameters). Edge colors indicate correlation directions: red (positive correlation), blue (negative correlation), gray (origin-specific association). The thickness of edges reflects the strength of correlations (**F**).

FAPROTAX predicted chemoheterotrophy and aerobic chemoheterotrophy as the dominant ecological functions (Figure 5A) [30]. The results showed that chemoheterotrophy and aerobic chemoheterotrophy were the predominant functions of microorganisms on the surface of ML from different origins, with relative abundances exceeding 35%. These functional profiles display correspondences to the basic survival of microbial communities in the harsh phyllosphere environment [31]. Notably, the GD group showed a significantly higher abundance of fermentation-related functions than the other two groups. This pattern parallels the high abundance and activity of lactic acid bacteria in this region and displays correspondences to shifts in flavor-precursor transformation. Subsequently, KEGG metabolic pathways were predicted using PICRUSt2 (Figure 5B). At KEGG level 1, metabolism was the most abundant functional category [32]. At KEGG level 3, pathways related to environmental adaptation, such as ABC transporters and two-component systems, were highly represented across samples from all three origins (Figure 5E). Differential analysis further revealed origin-specific metabolic preferences. The GD group showed greater predicted potential for “biosynthesis of secondary metabolites” and “phenylpropanoid biosynthesis”, which may provide a functional basis for the accumulation of polyphenols and flavonoids in this group. The YN group was significantly enriched in pathways related to “carbon metabolism” and “butanoate metabolism”. This functional profile mirrors the degradation of complex carbon sources on the leaf surface alongside small-molecule aroma-related metabolic events and shows concurrence with the superior aroma score of the YN samples in the sensory evaluation.

### 3.10. Environmental and Microbial Associations with Quality Differences

To further elucidate the complex relationships among environmental factors, epiphytic microorganisms, and quality attributes of ML samples, RDA was performed.

#### 3.10.1. Environmental Effects on Microbial Community Structure

Based on the ecological parameters listed in Table 1, RDA analysis was carried out to evaluate the associations of environmental factors with epiphytic microbial communities (Figure 5C). RDA1 and RDA2 jointly explained 79.38% of the variation in microbial community composition, accounting for 51.50% and 27.88%, respectively. Humidity and temperature were strongly positively associated with the GD samples and were closely related to the distribution of genera such as Enterobacter and Acinetobacter. The YN samples were mainly associated with altitude and sunshine, which was consistent with the possibility that the unique microclimate in this region selected for specific epiphytic microbial taxa. These findings indicate that region-specific epiphytic microbial community structures on ML samples are intertwined with local environmental conditions, with manifest linkages to temperature, humidity, light exposure, and precipitation.

#### 3.10.2. Associations Between Microorganisms and Metabolic Quality Traits

RDA integrating key differential genera, quality indicators, and characteristic metabolites was used to explore the chemical basis by which microorganisms may contribute to quality formation (Figure 5D). RDA1 and RDA2 together explained 77.61% of the total variation. GD samples clustered with TPC, TFC, FRAP, L-limonene, and p-cymene, and several genera showed vectors in the same direction. These patterns indicate covariance rather than a demonstrated microbial mechanism. This result supports the predicted enrichment of the “biosynthesis of secondary metabolites” pathway described in Section 3.9.

Regarding flavor-related compounds, α-phellandrene and dimethyl sulfide were associated with the GX and YN samples. Genera such as Methylobacterium showed positive associations with these compounds. Such observations display reciprocal ties to the profiles of characteristic flavor compounds in ML for specific epiphytic microorganisms.

### 3.11. Integrated Correlation Network of Quality Formation

To further explore geography-dependent associations related to quality formation in ML, a multilayer integrated correlation network was constructed (Figure 5F). The network illustrated associations among geographical origin, microbial patterns, metabolites, and quality traits.

The GD pathway was characterized by a microbial association pattern linked to high antioxidant activity. The GD samples were closely associated with marker taxa such as Lactobacillus and Bacilli-related taxa, which were strongly associated with the “biosynthesis of secondary metabolites” pathway. This pathway was further associated with the accumulation of key terpenoid compounds, including p-cymene and L-limonene. These characteristic metabolites showed strong positive correlations with antioxidant indices, including ABTS, DPPH, and FRAP, as well as TPC and TFC, providing a plausible biological basis for the strong antioxidant capacity of GD samples.

The YN pathway was mainly associated with superior sensory flavor quality. The YN samples were associated with specific microbial taxa, such as Bacteroidaceae and Lachnospiraceae, which were linked to carbon metabolism pathways. This pathway was associated with the formation of aromatic compounds such as α-phellandrene, styrene, and ethylbenzene. These compounds were positively correlated with aroma, taste, and infusion redness (a*) in the sensory evaluation, which may help explain the superior sensory performance of the YN samples.

The GX pathway was characterized by a microbial association pattern related to balanced flavor traits. The GX samples were marked by taxa such as Klebsiella, which were mainly associated with compounds such as dimethyl sulfide through the arginine and proline metabolism pathway, and were further linked to physical attributes such as infusion lightness (L*).

Not all epiphytic bacteria detected in this study are necessarily involved in aroma formation. Although RDA and correlation network analyses revealed associations between several bacterial taxa and volatile compounds, these relationships do not establish a direct metabolic contribution. The volatile profile of *Microcos paniculata* L. leaves may reflect both endogenous plant metabolism and microbial activity on the leaf surface. Terpenes such as limonene, p-cymene, and α-phellandrene may originate from plant metabolism, whereas sulfur-containing compounds such as dimethyl sulfide may also be influenced by microbial activity. However, their specific sources cannot be determined from the present data. Therefore, the observed associations should be interpreted as potential links between epiphytic bacterial communities and volatile composition rather than evidence of microbial production of individual aroma compounds. Further isolation and functional characterization of the associated bacterial strains are needed to clarify their roles in aroma formation.

## 4. Conclusions

This study revealed clear geographical differences in the quality characteristics of *Microcos paniculata* L. Guangdong samples showed higher total phenolic and flavonoid contents and higher antioxidant capacity, whereas Yunnan samples exhibited better sensory flavor, especially in aroma and taste. Metabolomic analysis identified 133 volatile compounds, among which p-cymene, L-limonene, α-phellandrene, dimethyl sulfide, and ethylbenzene were associated with origin discrimination and quality variation. 16S rRNA sequencing showed distinct epiphytic microbial communities among regions, with key taxa closely associated with characteristic metabolites and quality traits. Functional prediction, RDA, and correlation network analysis further uncover close couplings between environmental factors and microbial composition as well as metabolic pathways and establish observable linkages to flavor and antioxidant properties. These findings provide a foodomics-based basis for understanding geographical quality variation in ML and offer potential markers for origin traceability and quality evaluation. Further validation is needed to clarify the causal roles of specific microorganisms in flavor and bioactive compound formation.

## Figures and Tables

**Table 1:** Geographical and ecological characteristics of *Microcos paniculata* L. production regions.

Name	Place	Longitude/°E	Latitude/°N	Annual Temperature Difference/°C	Annual Sunshine Duration/h	Annual Precipitation/mm	Average Humidity/%	Average Altitude/m
GD	Guangdong Province	113°30′ E	23°24′ N	22	1800	1790	80	20
GX	Guangxi Province	108°21′ E	22°49′ N	21	1550	1540	83	75
YN	Yunnan Province	102°49′ E	24°53′ N	16	2100	1100	75	2000

## Data Availability

The original contributions presented in this study are included in the article/Supplementary Materials. Further inquiries can be directed to the corresponding authors.

## Supplementary Materials

The following supporting information can be downloaded at: https://www.mdpi.com/article/10.3390/foods15203744/s1, Table S1 The antioxidant properties (ABTS, DPPH, FRAP) and the contents of total phenols and total flavonoids in six different regions; Table S2 The color difference values of ML infusion among different regions; Table S3 The Sensor Performance Description; Table S4 A total of 133 metabolites were identified from ML; Table S5 Informed Consent Form for Sensory Evaluation Study; Figure S1: OPLS-DA Plot of GD vs. GX E-nose (A); OPLS-DA Plot of GD vs. YN E-nose (B); OPLS-DA Plot of GX vs. YN E-nose (C); Permutation Plot of GD vs. GX E-nose (D); Permutation Plot of GD vs. YN E-nose (E); Permutation Plot of GX vs. YN E-nose (F); Figure S2: Bar chart of the content of 12 key differential compounds (A); Hierarchical clustering heatmap of differential compounds between GD and GX samples (B); Hierarchical clustering heatmap of differential compounds between GX and YN samples (C); Hierarchical clustering heatmap of differential compounds between GD and YN samples (D).

## Abbreviations

The following abbreviations are used in this manuscript:

ML	*Microcos paniculata* L.
GD	Guangdong
GX	Guangxi
YN	Yunnan
