# Supplementary material for: Integrated Volatile Metabolomics and Bacterial Community Profiling Identify Potential Quality Markers for Geographical Discrimination of Microcos paniculata L. Herbal Tea
Source: Foods. 2026 Oct 21;15(20):3744. doi: 10.3390/foods15203744 (PMC13704147; doi:10.3390/foods15203744)
Supplement: Supplementary file 1 [file foods-15-03744-s001.zip › foods-4571159-supplementary.pdf]

Integrated volatile metabolomics and bacterial community profiling identify potential quality markers for geographical discrimination of Microcos paniculata L. herbal tea

Yihang He[a,†], Pu Xu[a,†], Jianing Zhou[a], Jiayi Zheng[a], Riyan Yuchi[a], Xintong Wang[a], Ning Ma[a], Binbin Wei[a,*] and Song Cang [b,*]

[a]School of Pharmacy, China Medical University, No. 77 Puhe Road, Shenyang 110122, China

[b]Department of Sanitary Inspection, School of Public Health, Shenyang Medical College, No. 146 North Huanghe Street, Shenyang, Liaoning 110034, China

[*]Correspondence: wbb3127@163.com (B.W.); liuzhanfu@symc.edu.cn (Z.L.); cangsong@symc.edu.cn (S.C.)

[†]These authors contributed equally to this work.

ABTS radical scavenging activity

In brief, an ABTS working solution was formulated by reacting 7 mmol/L ABTS with an equal volume of 2.45 mmol/L potassium persulfate. The resultant mixture was diluted and then allowed to incubate for 12 h in the dark at room temperature to attain a stable absorbance of 0.70 ± 0.02 at 734 nm. To perform the assay, 200 μL of each diluted sample was combined with 2.8 mL of the prepared ABTS solution. After 6 min of reaction at 25 °C in the dark, the absorbance was measured at 734 nm. The antioxidant capacity was quantified using a Trolox standard curve and expressed as micromolar Trolox equivalents per gram (μmol TE/g).

DPPH radical scavenging activity

In brief, 0.5 mL of the suitably diluted sample solution was combined with 2.5

mL of a freshly prepared 80 μM DPPH ethanol solution. The resultant mixture was incubated in the dark for 2 h. Absorbance was subsequently measured at 515 nm using a microplate reader. The scavenging capacity was quantified using a Trolox standard calibration curve and expressed as micromolar Trolox equivalents per gram (μmol TE/g).

FRAP detection

In brief, TPTZ (0.31 g) was dissolved in 100 mL of 40 mM HCl to prepare the TPTZ solution. The FRAP working solution was formulated by mixing the TPTZ solution, 20 mM $FeCl_3 \cdot 6H_2O$ aqueous solution, and acetate buffer (0.3 M, pH 3.6) at a volume ratio of 1:1:10. The resulting solution was then incubated at 37 °C. Subsequently, 200 μL of the appropriately diluted sample was added to 2.8 mL of the preheated FRAP working solution and the mixture was incubated at room temperature for 30 min. Absorbance was measured at 593 nm. The reducing power was quantified using a Trolox standard calibration curve and expressed as micromolar Trolox equivalents per gram (μmol TE/g).

TPC detection

In brief, a freshly diluted Folin–Ciocalteu reagent (1:9, v/v) was employed. Specifically, 2 mL of the diluted reagent was mixed with 1 mL of the appropriately diluted sample extract, followed by the addition of 2 mL of sodium carbonate solution (75 g/L). The resulting mixture was vortexed for 1 min and incubated in the dark at room temperature for 40 min. Absorbance was then measured at 760 nm using a microplate reader. The phenolic content was quantified via gallic acid calibration and

expressed in gallic acid equivalents (GAE).

TFC detection

In brief, 0.8 mL of the appropriately diluted sample extract was mixed with 0.3 mL of 5% sodium nitrite solution and incubated in the dark for 6 min. Then, 0.3 mL of 10% aluminum nitrate solution was added, and the mixture was incubated again in the dark for another 6 min. Thereafter, 4 mL of 1 M sodium hydroxide solution was incorporated, and the volume was adjusted to 10 mL with 60% ethanol. The resulting mixture was kept in the dark at ambient temperature for 10 min, and the absorbance was measured at 510 nm using a microplate reader. The flavonoid content was quantified using a rutin standard calibration curve and expressed as rutin equivalents (RE).

Group	ABTS	DPPH	FRAP	TPC	TFC
GD1	2.962	4.191	2.142	50.522	159.951
GD2	2.983	4.282	2.262	50.962	161.852
GD3	3.201	4.524	2.544	52.584	165.903
GD4	3.003	4.382	2.413	51.203	165.613
GD5	3.104	4.423	2.494	51.964	165.164
GD6	3.303	4.644	2.663	53.603	166.013
GX1	2.997	4.221	1.291	33.061	156.354
GX2	2.952	4.000	0.896	13.944	13.623
GX3	2.972	4.080	0.900	15.174	45.409
GX4	2.982	4.124	0.959	19.865	52.175
GX5	2.989	4.169	1.063	25.327	90.266

GX6	2.994	4.196	1.187	29.758	128.947
YN1	2.917	4.017	0.309	11.650	27.986
YN2	2.932	4.045	0.413	13.013	43.346
YN3	2.941	4.056	0.640	17.169	57.750
YN4	2.942	4.059	0.720	15.806	64.980
YN5	2.946	4.067	0.758	20.366	80.950
YN6	2.960	4.152	1.071	24.151	110.671

Table S1 The antioxidant properties (ABTS, DPPH, FRAP) and the contents of total phenols and total flavonoids in six different regions.

Name	GD	GX	YN
GD	0		
GX	4.18	0	
YN	2.13	2.04	0

Table S2 The color difference values of ML infusion among different regions.

No.	Sensors	Sensor sensitives
1	W1C	Benzene
2	W5S	Nitrogen oxides
3	W3C	Ammonia
4	W6S	Hydrogen
5	W5C	Alkane aromatic components
6	W1S	Alkanes(Sensitive to short-chain alkanes)
7	W1W	Inorganic Sulfur Compounds

8	W2S	Alcohols, Ethers, Aldehydes, and Ketones
9	W2W	Aromatic and Organic Sulfur Compounds
10	W3S	Alkanes(Sensitive to long-chain alkanes)

Table S3 The Sensor Performance Description.

**Compound name**	**CAS**	**Formula (mol ion)**	**HMDB**	**Class**
1,8-Cineole	470-82-6	C10H18O	HMDB0004472	alcohols and derivatives
2,4-Hexadienal	142-83-6	C6H8O	HMDB0034729	alcohols and derivatives
Acetic acid	64-19-7	C2H4O2	HMDB0000042	alcohols and derivatives
Benzaldehyde	100-52-7	C7H6O	HDMB0006115	alcohols and derivatives
Campholenal	4501-58-0	C10H16O	HMDB0034973	alcohols and derivatives
Methyl atropate	1865-29-8	C10H10O2	——	alcohols and derivatives
p-Tolylmethylcarbinol	536-50-5	C9H12O	——	alcohols and derivatives
3,5-Nonadien-7-yn-2-ol, (E,E)-	43142-43-4	C9H12O	——	alcohols and derivatives
Acetaldehyde	75-07-0	C2H4O	HMDB0000990	alcohols and derivatives
Benzenemethanol	100-51-6	C7H8O	HMDB0003119	alcohols and derivatives
3-Furaldehyde	498-60-2	C5H4O2	——	alcohols and derivatives
Sabinene	432-25-7	C10H16O	HMDB0041011	alcohols and derivatives
2,2-dimethylvaleraldehyde	14250-88-5	C7H14O	——	alcohols and derivatives
Sulcatone	75-56-9	C3H6O	HMDB0031558	alcohols and derivatives
2-Furanmethanol	98-00-0	C5H6O2	HMDB0013742	alcohols and derivatives
cis-Piperitol	16721-38-3	C10H18O	——	alcohols and derivatives

Propanal	123-38-6	C_3H_6O	HMDB0003366	alcohols and derivatives
Propanal, 2-methyl-	78-84-2	C_4H_8O	HMDB0031243	alcohols and derivatives
Pentanal, 2-methyl-	123-15-9	$C_6H_{12}O$	HMDB0031578	alcohols and derivatives
Oxirane, ethyl-	106-88-7	C_4H_8O	——	alcohols and derivatives
1,1-Dimethylcyclopropane	1630-94-0	C_5H_{10}	——	alkanes and derivatives
Cyclopropylacetylene	6746-94-7	C_5H_6	——	alkanes and derivatives
Benzene, ethyl-	100-41-4	C_8H_{10}	HMDB0059905	aromatic compounds
Tropilidene	544-25-2	C_7H_8	——	aromatic compounds
Styrene	100-42-5	C_8H_8	HMDB0034240	aromatic compounds
Benzene, methyl-	108-88-3	C_7H_8	HMDB0034168	aromatic compounds
Eugenol	97-53-0	$C_{10}H_{12}O_2$	HMDB0005809	aromatic compounds
1,4-Benzenediol	123-31-9	$C_6H_6O_2$	HMDB0002434	aromatic compounds
xylene	1330-20-7	C_8H_{10}	——	aromatic compounds
Benzene, 1,3-dimethyl-	108-38-3	C_8H_{10}	——	aromatic compounds
trans-anethole	4180-23-8	$C_{10}H_{12}O$	HMDB0030837	aromatic compounds
2-Methoxy-4-vinylphenol	7786-61-0	$C_9H_{10}O_2$	HMDB0013744	aromatic compounds
Phenol, 4-methoxy-	150-76-5	$C_7H_8O_2$	HMDB0029696	aromatic compounds
Methyl salicylate	119-36-8	$C_8H_8O_3$	HMDB0034172	aromatic compounds
Butylated hydroxytoluene	128-37-0	$C_{15}H_{24}O$	HMDB0033826	aromatic compounds
3-thujene	3479-89-8	$C_{10}H_{14}$	——	aromatic compounds
Phenol, 4-ethyl-2-methyl-	2219-73-0	$C_9H_{12}O$	HMDB0038984	aromatic compounds
Benzene, 1,2-dimethoxy-	91-16-7	$C_8H_{10}O_2$	HMDB0032139	aromatic compounds

Precocene I	17598-02-6	C12H14O2	——	aromatic compounds
2-tert-Butylphenol	88-18-6	C10H14O	——	aromatic compounds
Benzene	71-43-2	C6H6	HMDB0001505	aromatic compounds
Benzene, 1,3-diethyl-	141-93-5	C10H14	——	aromatic compounds
Estragole	140-67-0	C10H12O	HMDB0034121	aromatic compounds
Benzene, 1,2-diethyl-	135-01-3	C10H14	——	aromatic compounds,
alpha-Curcumene	644-30-4	C15H22	——	aromatic compounds,
Isopulegone	88-69-7	C9H12O	HMDB0032029	aromatic compounds,
Phenol, 3-ethyl-5-methyl-	698-71-5	C9H12O	HMDB0038985	aromatic compounds,
Benzene, 1,4-diethyl-	105-05-5	C10H14	——	aromatic compounds,
4-Vinyl-o-xylene	27831-13-6	C10H12	——	aromatic compounds,
Phenol	108-95-2	C6H6O	HMDB0000228	aromatic compounds,
2-sec-Butylphenol	89-72-5	C10H14O	——	aromatic compounds,
2,2-Dimethylchroman	1198-96-5	C11H14O	——	aromatic compounds,
Paeonal	552-41-0	C9H10O3	——	ketones
Acetone	67-64-1	C3H6O	HMDB0001659	ketones
2-Pentanone	107-87-9	C5H10O	HMDB0034235	ketones
Seudenone	1193-18-6	C7H10O	HMDB0031541	ketones
4-Methyltropone	690233-50-2	C8H8O	——	ketones
(Z)-Tagetone	3588-18-9	C10H16O	——	ketones
Furfuryl alcohol	89-80-5	C10H18O	——	ketones
2-Heptanone	110-43-0	C7H14O	HMDB0003671	ketones
(+)-2-Bornanone	464-49-	C10H16O	HMDB00	ketones

	3		59838	
2-Octanone	111-13-7	C8H16O	HMDB0031294	ketones
6-Octen-2-one, (Z)-	74810-53-0	C8H14O	——	ketones
Sulcatone	110-93-0	C8H14O	HMDB0035915	ketones
2-Butanone, 3-methyl-	563-80-4	C5H10O	——	ketones
Cyclohexanone	108-94-1	C6H10O	HMDB0003315	ketones
p-menth-8-en-3-one	529-00-0	C10H16O	——	ketones
Cyclopentene, 3-methyl-	1120-62-3	C6H10	HMDB0031544	olefins and cycloolefins
5,7-Dodecadiene, (E,Z)-	21293-04-9	C12H22	——	olefins and cycloolefins
1,4-Heptadiene	5675-22-9	C7H12	——	olefins and cycloolefins
Pentane, 3-methylene-	760-21-4	C6H12	——	olefins and cycloolefins
trans-.beta.-Ocimene	3779-61-1	C10H16	HMDB0030089	olefins and cycloolefins
2-Butene, 2,3-dimethyl-	563-79-1	C6H12	——	olefins and cycloolefins
laurolene	473-91-6	C8H14	——	olefins and cycloolefins
Cyclopentene, 1-methyl-	693-89-0	C6H10	——	olefins and cycloolefins
2,5-Dihydrotoluene	4313-57-9	C7H10	——	olefins and cycloolefins
Cyclopropane, (R,R)-1-((Z),(Z)-hexa-1',3'-dienyl)-2-ethenyl-	77210-41-4	C11H16	——	olefins and cycloolefins
Bicyclo[3.2.0]hept-6-ene	4927/3/1	C7H10	——	olefins and cycloolefins
Methylcyclohexadiene	1489-56-1	C7H10	HMDB0031532	olefins and cycloolefins
alpha-Pyronene	514-94-3	C10H16	——	olefins and cycloolefins
1,2-Dimethylcyclopentadiene	4784-86-5	C7H10	——	olefins and cycloolefins
Ethene, methoxy-	107-25-5	C3H6O	——	olefins and cycloolefins

1,4-Pentadiene, 3-methyl-	1115-08-8	C6H10	——	olefins and cycloolefins
2-Methyl-7-exo-vinylbicyclo[4.2.0]oct-1(2)-ene	107914-89-6	C11H16	——	olefins and cycloolefins
2-Buten-1-ol, (E)-	504-61-0	C4H8O	——	olefins and cycloolefins
1-Heptene, 3-methoxy-	14093-58-4	C8H16O	——	olefins and cycloolefins
2-Butene, 2-methyl-	513-35-9	C5H10	——	olefins and cycloolefins
1-Propene, 2-methyl-	115-11-7	C4H8	——	olefins and cycloolefins
Melonal	106-72-9	C9H16O	——	olefins and cycloolefins
Cyclohexene, 1-(1-propynyl)-	1655-05-6	C9H12	——	olefins and cycloolefins
exo-7-(2-Propenyl)bicyclo[4.2.0]oct-1(2)-ene	-	C11H16	——	olefins and cycloolefins
Biisopropenyl	513-81-5	C6H10	——	olefins and cycloolefins
1,2-Cyclooctadiene	7124-40-5	C8H12	——	olefins and cycloolefins
Bicyclo[3.1.0]hexane, 6-methylene-	54211-16-4	C7H10	——	olefins and cycloolefins
1-methyl-3-isobutenylcyclopentane	75873-01-7	C10H18	——	olefins and cycloolefins
Cycloocta-1,4-diene	16327-22-3	C8H12	——	olefins and cycloolefins
Cubenene	29837-12-5	C15H24	——	olefins and cycloolefins
Spiro[2,4]hepta-4,6-diene	765-46-8	C7H8	——	olefins and cycloolefins
2-Acetyl-5-methylfuran	1193-79-9	C7H8O2	HMDB0035226	oxygen heterocycle
Furan, 2-ethyl-	3208-16-0	C6H8O	HMDB0040587	oxygen heterocycle
Safrole	94-59-7	C10H10O2	HMDB0033591	oxygen heterocycle
Furan, 2-pentyl-	3777-69-3	C9H14O	HMDB0013824	oxygen heterocycle
Furan, 2-methyl-	534-22-5	C5H6O	HMDB0013749	oxygen heterocycle
Dihydroedulan	41678-32-4	C13H22O	——	oxygen heterocycle

Spiro[4.4]nona-1,3-diene, 1,2-dimethyl-	872629-13-5	C11H16	——	spiro compounds
Spiro[2.4]heptane, 1,5-dimethyl-6-methylene-	62238-24-8	C10H16	——	spiro compounds
α-Pyronene	185-65-9	C8H14	——	spiro compounds
3,4-Dimethylstyrene	54211-14-2	C8H12	——	spiro compounds
2-Allylbicyclo[2.2.1]heptane	2633-80-9	C10H16	——	spiro compounds
Dimethyl sulfide	75-18-3	C2H6S	HMDB0002303	sulfur-containing compound
Ethanethiol	1975/8/1	C2H6S	——	sulfur-containing compound
alpha.-Phellandrene	99-83-2	C10H16	HMDB0035850	terpenes
p-Cymene	99-87-6	C10H14	HMDB0005805	terpenes
(-)-Limonene	5989-54-8	C10H16	HMDB0004321	terpenes
cis-Muurola-4(15),5-diene	157477-72-0	C15H24	——	terpenes
D-Limonene	5989-27-5	C10H16	HMDB0003375	terpenes
cis-muurola-3,5-diene	-	C15H24	——	terpenes
p-mentha-1(7),3-diene	99-84-3	C10H16	HMDB0036997	terpenes
.alpha-thujene	353313	C10H16	——	terpenes
.gamma.-Terpinene	99-85-4	C10H16	HMDB0005806	terpenes
.alpha.-Cubebene	17699-14-8	C15H24	HMDB0036413	terpenes
(-)-alpha-Pinene	7785-26-4	C10H16	HMDB0035658	terpenes
Germacrene D	23986-74-5	C15H24	——	terpenes
safranal	116-26-7	C10H14O	HMDB0036061	terpenes
Bicyclo[3.1.0]hexane, 4-methylene-1-(1-methylethyl)-	3387-41-5	C10H16	——	terpenes
trans-Calamenene	73209-42-4	C15H22	——	terpenes
Isoterpinolene	586-63-0	C10H16	——	terpenes

Terpilene	99-86-5	C10H16	HMDB0036995	terpenes
Isoprene	78-79-5	C5H8	——	terpenes
Delta-Guaiene	3691-11-0	C15H24	HMDB0036444	terpenes
(+)-Eremophilene	10219-75-7	C15H24	——	terpenes
.alpha.-Muurolene	31983-22-9	C15H24	——	terpenes
.alpha.-Cyperene	473-08-5	C15H22O	HMDB0037061	terpenes

**Compound name**	**CAS**	**Formula (mol ion)**	**HMDB**	**Class**
1,8-Cineole	470-82-6	C10H18O	HMDB0004472	alcohols and derivatives
2,4-Hexadienal	142-83-6	C6H8O	HMDB0034729	alcohols and derivatives
Acetic acid	64-19-7	C2H4O2	HMDB0000042	alcohols and derivatives
Benzaldehyde	100-52-7	C7H6O	HDMB0006115	alcohols and derivatives
(1,2,2-trimethyl-3-cyclopenten-1-yl)acetaldehyde	-	C10H16O	——	alcohols and derivatives
Methyl 2-phenyl-prop-2-enoate	1865-29-8	C10H10O2	——	alcohols and derivatives
Benzenemethanol, .alpha.,4-dimethyl-	536-50-5	C9H12O	——	alcohols and derivatives
3,5-Nonadien-7-yn-2-ol, (E,E)-	43142-43-4	C9H12O	——	alcohols and derivatives
Acetaldehyde	75-07-0	C2H4O	HMDB0000990	alcohols and derivatives
Benzenemethanol	100-51-6	C7H8O	HMDB0003119	alcohols and derivatives
3-Furaldehyde	498-60-2	C5H4O2	——	alcohols and derivatives
1-Cyclohexene-1-carboxaldehyde, 2,6,6-trimethyl-	432-25-7	C10H16O	HMDB0041011	alcohols and derivatives
Pentanal, 2,2-dimethyl-	14250-88-5	C7H14O	——	alcohols and derivatives
Oxirane, methyl-	75-56-9	C3H6O	HMDB0031558	alcohols and derivatives
2-Furanmethanol	98-00-0	C5H6O2	HMDB0013742	alcohols and derivatives
2-Cyclohexen-1-ol,	16721-3	C10H18O	——	alcohols and

3-methyl-6-(1-methylethyl)-, cis-	8-3			derivatives
Propanal	123-38-6	C3H6O	HMDB0003366	alcohols and derivatives
Propanal, 2-methyl-	78-84-2	C4H8O	HMDB0031243	alcohols and derivatives
Pentanal, 2-methyl-	123-15-9	C6H12O	HMDB0031578	alcohols and derivatives
Oxirane, ethyl-	106-88-7	C4H8O	——	alcohols and derivatives
Cyclopropane, 1,1-dimethyl-	1630-94-0	C5H10	——	alkanes and derivatives
Cyclopropylacetylene	6746-94-7	C5H6	——	alkanes and derivatives
Benzene, ethyl-	100-41-4	C8H10	HMDB0059905	aromatic compounds
1,3,5-Cycloheptatriene	544-25-2	C7H8	——	aromatic compounds
Styrene	100-42-5	C8H8	HMDB0034240	aromatic compounds
Benzene, methyl-	108-88-3	C7H8	HMDB0034168	aromatic compounds
Phenol, 2-methoxy-4-(2-propenyl)-	97-53-0	C10H12O2	HMDB0005809	aromatic compounds
1,4-Benzenediol	123-31-9	C6H6O2	HMDB0002434	aromatic compounds
xylene	1330-20-7	C8H10	——	aromatic compounds
Benzene, 1,3-dimethyl-	108-38-3	C8H10	——	aromatic compounds
trans-anethole	4180-23-8	C10H12O	HMDB0030837	aromatic compounds
2-Methoxy-4-vinylphenol	7786-61-0	C9H10O2	HMDB0013744	aromatic compounds
Phenol, 4-methoxy-	150-76-5	C7H8O2	HMDB0029696	aromatic compounds
Methyl salicylate	119-36-8	C8H8O3	HMDB0034172	aromatic compounds
Phenol, 2,6-bis(1,1-dimethylethyl)-4-methyl-	128-37-0	C15H24O	HMDB0033826	aromatic compounds
1,3,5-Cycloheptatriene, 3,7,7-trimethyl-	3479-89-8	C10H14	——	aromatic compounds
Phenol, 4-ethyl-2-methyl-	2219-73-0	C9H12O	HMDB0038984	aromatic compounds
Benzene, 1,2-dimethoxy-	91-16-7	C8H10O2	HMDB00	aromatic

			32139	compounds
Precocene I	17598-02-6	C12H14O2	——	aromatic compounds
Phenol, 2-(1,1-dimethylethyl)-	88-18-6	C10H14O	——	aromatic compounds
Benzene	71-43-2	C6H6	HMDB0001505	aromatic compounds
Benzene, 1,3-diethyl-	141-93-5	C10H14	——	aromatic compounds
Estragole	140-67-0	C10H12O	HMDB0034121	aromatic compounds
Benzene, 1,2-diethyl-	135-01-3	C10H14	——	aromatic compounds,
Benzene, 1-(1,5-dimethyl-4-hexenyl)-4-methyl-	644-30-4	C15H22	——	aromatic compounds,
Phenol, 2-(1-methylethyl)-	88-69-7	C9H12O	HMDB0032029	aromatic compounds,
Phenol, 3-ethyl-5-methyl-	698-71-5	C9H12O	HMDB0038985	aromatic compounds,
Benzene, 1,4-diethyl-	105-05-5	C10H14	——	aromatic compounds,
Benzene, 4-ethenyl-1,2-dimethyl-	27831-13-6	C10H12	——	aromatic compounds,
Phenol	108-95-2	C6H6O	HMDB0000228	aromatic compounds,
Phenol, 2-(1-methylpropyl)-	89-72-5	C10H14O	——	aromatic compounds,
2H-1-Benzopyran, 3,4-dihydro-2,2-dimethyl-	1198-96-5	C11H14O	——	aromatic compounds,
Ethanone, 1-(2-hydroxy-4-methoxyphenyl)-	552-41-0	C9H10O3	——	ketones
Acetone	67-64-1	C3H6O	HMDB0001659	ketones
2-Pentanone	107-87-9	C5H10O	HMDB0034235	ketones
2-Cyclohexen-1-one, 3-methyl-	1193-18-6	C7H10O	HMDB0031541	ketones
2,4,6-Cycloheptatrien-1-one, 4-methyl-	690233-50-2	C8H8O	——	ketones
5,7-Octadien-4-one, 2,6-dimethyl-, (Z)-	3588-18-9	C10H16O	——	ketones
Cyclohexanone, 5-methyl-2-(1-methylethyl)-, trans-	89-80-5	C10H18O	——	ketones
2-Heptanone	110-43-	C7H14O	HMDB00	ketones

	0		03671	
(+)-2-Bornanone	464-49-3	C10H16O	HMDB0059838	ketones
2-Octanone	111-13-7	C8H16O	HMDB0031294	ketones
6-Octen-2-one, (Z)-	74810-53-0	C8H14O	——	ketones
5-Hepten-2-one, 6-methyl-	110-93-0	C8H14O	HMDB0035915	ketones
2-Butanone, 3-methyl-	563-80-4	C5H10O	——	ketones
Cyclohexanone	108-94-1	C6H10O	HMDB0003315	ketones
Cyclohexanone, 5-methyl-2-(1-methylethenyl)-	529-00-0	C10H16O	——	ketones
Cyclopentene, 3-methyl-	1120-62-3	C6H10	HMDB0031544	olefins and cycloolefins
5,7-Dodecadiene, (E,Z)-	21293-04-9	C12H22	——	olefins and cycloolefins
1,4-Heptadiene	5675-22-9	C7H12	——	olefins and cycloolefins
Pentane, 3-methylene-	760-21-4	C6H12	——	olefins and cycloolefins
trans-.beta.-Ocimene	3779-61-1	C10H16	HMDB0030089	olefins and cycloolefins
2-Butene, 2,3-dimethyl-	563-79-1	C6H12	——	olefins and cycloolefins
Cyclopentene, 1,2,3-trimethyl-	473-91-6	C8H14	——	olefins and cycloolefins
Cyclopentene, 1-methyl-	693-89-0	C6H10	——	olefins and cycloolefins
1,4-Cyclohexadiene, 1-methyl-	4313-57-9	C7H10	——	olefins and cycloolefins
Cyclopropane, (R,R)-1-((Z),(Z)-hexa-1',3'-dienyl)-2-ethenyl-	77210-41-4	C11H16	——	olefins and cycloolefins
Bicyclo[3.2.0]hept-6-ene	4927/3/1	C7H10	——	olefins and cycloolefins
1-Methylcyclohexa-1,3-diene	1489-56-1	C7H10	HMDB0031532	olefins and cycloolefins
1,3-Cyclohexadiene, 1,5,5,6-tetramethyl-	514-94-3	C10H16	——	olefins and cycloolefins
1,3-Cyclopentadiene, 1,2-dimethyl-	4784-86-5	C7H10	——	olefins and cycloolefins

Ethene, methoxy-	107-25-5	C_3H_6O	——	olefins and cycloolefins
1,4-Pentadiene, 3-methyl-	1115-08-8	C_6H_{10}	——	olefins and cycloolefins
2-Methyl-7-exo-vinylbicyclo[4.2.0]oct-1(2)-ene	107914-89-6	$C_{11}H_{16}$	——	olefins and cycloolefins
2-Buten-1-ol, (E)-	504-61-0	C_4H_8O	——	olefins and cycloolefins
1-Heptene, 3-methoxy-	14093-58-4	$C_8H_{16}O$	——	olefins and cycloolefins
2-Butene, 2-methyl-	513-35-9	C_5H_{10}	——	olefins and cycloolefins
1-Propene, 2-methyl-	115-11-7	C_4H_8	——	olefins and cycloolefins
5-Heptenal, 2,6-dimethyl-	106-72-9	$C_9H_{16}O$	——	olefins and cycloolefins
Cyclohexene, 1-(1-propynyl)-	1655-05-6	C_9H_{12}	——	olefins and cycloolefins
exo-7-(2-Propenyl)bicyclo[4.2.0]oct-1(2)-ene	-	$C_{11}H_{16}$	——	olefins and cycloolefins
1,3-Butadiene, 2,3-dimethyl-	513-81-5	C_6H_{10}	——	olefins and cycloolefins
1,2-Cyclooctadiene	7124-40-5	C_8H_{12}	——	olefins and cycloolefins
Bicyclo[3.1.0]hexane, 6-methylene-	54211-16-4	C_7H_{10}	——	olefins and cycloolefins
Cyclopentane, 1-methyl-3-(2-methyl-1-propenyl)-	75873-01-7	$C_{10}H_{18}$	——	olefins and cycloolefins
1,4-Cyclooctadiene, (Z,Z)-	16327-22-3	C_8H_{12}	——	olefins and cycloolefins
Cubenene	29837-12-5	$C_{15}H_{24}$	——	olefins and cycloolefins
Spiro[2,4]hepta-4,6-diene	765-46-8	C_7H_8	——	olefins and cycloolefins
2-Acetyl-5-methylfuran	1193-79-9	$C_7H_8O_2$	HMDB0035226	oxygen heterocycle
Furan, 2-ethyl-	3208-16-0	C_6H_8O	HMDB0040587	oxygen heterocycle
1,3-Benzodioxole, 5-(2-propenyl)-	94-59-7	$C_{10}H_{10}O_2$	HMDB0033591	oxygen heterocycle
Furan, 2-pentyl-	3777-69-3	$C_9H_{14}O$	HMDB0013824	oxygen heterocycle
Furan, 2-methyl-	534-22-5	C_5H_6O	HMDB0013749	oxygen heterocycle

Dihydroedulan	41678-32-4	C13H22O	——	oxygen heterocycle
Spiro[4.4]nona-1,3-diene, 1,2-dimethyl-	872629-13-5	C11H16	——	spiro compounds
Spiro[2.4]heptane, 1,5-dimethyl-6-methylene-	62238-24-8	C10H16	——	spiro compounds
Spiro[2.5]octane	185-65-9	C8H14	——	spiro compounds
Bicyclo[4.1.0]heptane, 7-methylene-	54211-14-2	C8H12	——	spiro compounds
Bicyclo[2.2.1]heptane, 2-(2-propenyl)-	2633-80-9	C10H16	——	spiro compounds
Dimethyl sulfide	75-18-3	C2H6S	HMDB0002303	sulfur-containing compound
Ethanethiol	1975/8/1	C2H6S	——	sulfur-containing compound
alpha.-Phellandrene	99-83-2	C10H16	HMDB0035850	terpenes
p-Cymene	99-87-6	C10H14	HMDB0005805	terpenes
Cyclohexene, 1-methyl-4-(1-methylethenyl)-, (S)-	5989-54-8	C10H16	HMDB0004321	terpenes
cis-Muurola-4(15),5-diene	157477-72-0	C15H24	——	terpenes
D-Limonene	5989-27-5	C10H16	HMDB0003375	terpenes
cis-muurola-3,5-diene	-	C15H24	——	terpenes
Cyclohexene, 4-methylene-1-(1-methylethyl)-	99-84-3	C10H16	HMDB0036997	terpenes
Bicyclo[3.1.0]hex-2-ene, 2-methyl-5-(1-methylethyl)-	353313	C10H16	——	terpenes
.gamma.-Terpinene	99-85-4	C10H16	HMDB0005806	terpenes
.alpha.-Cubebene	17699-14-8	C15H24	HMDB0036413	terpenes
(1S)-2,6,6-Trimethylbicyclo[3.1.1]hept-2-ene	7785-26-4	C10H16	HMDB0035658	terpenes
Germacrene D	23986-74-5	C15H24	——	terpenes
1,3-Cyclohexadiene-1-carboxaldehyde, 2,6,6-trimethyl-	116-26-7	C10H14O	HMDB0036061	terpenes
Bicyclo[3.1.0]hexane, 4-methylene-1-(1-methylethyl)-	3387-41-5	C10H16	——	terpenes
trans-Calamenene	73209-4	C15H22	——	terpenes

	2-4			
Cyclohexene, 3-methyl-6-(1-methylethylidene)-	586-63-0	C10H16	——	terpenes
1,3-Cyclohexadiene, 1-methyl-4-(1-methylethyl)-	99-86-5	C10H16	HMDB0036995	terpenes
Isoprene	78-79-5	C5H8	——	terpenes
Delta-Guaiene	3691-11-0	C15H24	HMDB0036444	terpenes
(+)-Eremophilene	10219-75-7	C15H24	——	terpenes
α-Muurolene	31983-22-9	C15H24	——	terpenes
α-Cyperene	473-08-5	C15H22O	HMDB0037061	terpenes

Table S4 A total of 133 metabolites were identified from ML.

Informed Consent Form for Sensory Evaluation Study

Project Title: Quantitative Descriptive Analysis of Microcos paniculata L. Tea Sensory Characteristics

Research Institution: China Medical University (Shenyang)

Dear Sir/Madam,

We cordially invite you to participate in this scientific research on the sensory characteristics of *Microcos paniculata L.* tea . Before you decide whether to participate, please read the following information carefully to understand the purpose, procedures, potential benefits and risks of this study, as well as your rights. If you have any questions, please feel free to ask our research staff at any time.

1. Purpose of the Research

This study aims to scientifically analyze and describe the flavor profiles of different

Microcos paniculata L. tea samples using professional sensory evaluation methods, to establish objective evaluation criteria for their sensory quality.

2. What Will You Be Asked To Do?

If you agree to participate, we will ask you to:

1.Attend Training: You will need to participate in approximately 5 days of training, about 1 hour per day. The training will cover learning to identify and quantify specific flavor attributes (e.g., sweetness, sourness) and practicing using a unified scoring scale.

2.Take an Assessment: After the training, you will need to pass a sensory test (including distinguishing and ranking samples) to ensure the consistency and reliability of the evaluation results.

3.Perform Formal Evaluations: During the formal evaluation sessions, you will taste a series of coded Microcos paniculata L. tea samples in a designated laboratory environment. For each sample, you will be required to chew for a few seconds and then expectorate it (swallowing is prohibited), and immediately record your perception of the intensity of various flavors on an electronic questionnaire. Each evaluation session will last approximately 45 minutes.

3. Potential Risks and Discomforts

1.Tasting samples may cause very minor, temporary oral discomfort. All Microcos paniculata L. tea samples are food-grade, with additive residues strictly below the limits set by Chinese national standards and are safe for consumption (though must be expectorated).

2.This study has excluded known common allergens; however, if you have a known allergy to Microcos paniculata L., please do not participate.

3.You might experience slight fatigue or decreased attention. To minimize this, we have scheduled mandatory breaks and palate cleansing procedures.

4. Potential Benefits

1.You will gain valuable experience in sensory evaluation training and deepen your knowledge of food sensory science methods.

2.Your research data will provide valuable baseline data for food science research.

3.You will receive appropriate compensation or a token of appreciation for your time and effort.

5. Your Rights

1.Your participation is entirely voluntary. Even after signing this consent form, you have the right to withdraw from the study at any time, for any reason, without any negative consequences to your relationship with the researchers.

2.All your personal information and evaluation data will be kept strictly confidential. Your identity will not be disclosed in any research reports or publications; all data will be analyzed and presented anonymously.

3.If you feel any discomfort during the study, you can pause or terminate the evaluation immediately.

6. Costs and Compensation

There are no costs to you for participating in this study. Upon successful completion of the training and all evaluation sessions, you will receive the agreed-upon

compensation.

7. Contact Information

If you have any questions, concerns, or encounter any problems during the study, please contact the principal investigator of this study using the following details:

Name: Yihang He

Contact Phone Number: +86 18201127670

E-mail: heyihang0302@outlook.com

Volunteer Statement

I have carefully read and understood the information provided above. All my questions have been answered satisfactorily. I voluntarily agree to participate in the research titled "Quantitative Descriptive Analysis of Microcos paniculata L. tea Sensory Characteristics" and commit to completing the training and evaluations according to the instructions of the research staff.

Volunteer Signature: Qiuue Luo Qingyi Jin

Yuxi Chen Pengfei Zhao Pengyu Bai

ziqi Yang

Date: 2025/3/28

Researcher Statement

I confirm that I have explained the purpose, procedures, risks, and rights involved in this study to the volunteer in detail and have answered all of their questions.

Researcher Signature: _______ Yihang He _____ Date: 2025/3/28

Table S5 Informed Consent Form for Sensory Evaluation Study

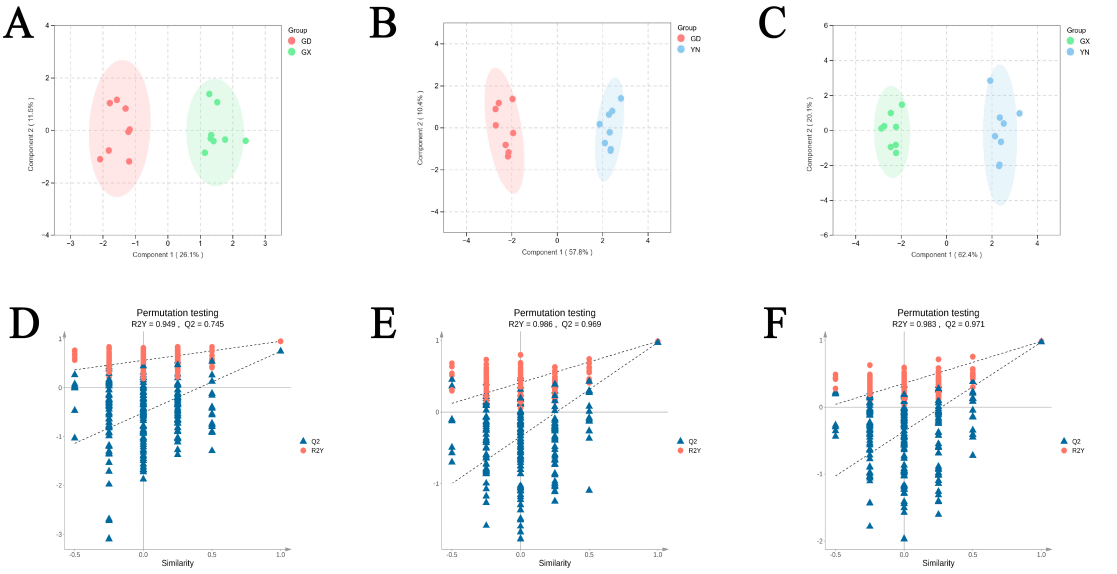

Fig S1: OPLS-DA Plot of GD vs. GX E-nose (A); OPLS-DA Plot of GD vs. YN E-nose (B); OPLS-DA Plot of GX vs. YN E-nose (C); Permutation Plot of GD vs. GX E-nose (D); Permutation Plot of GD vs. YN E-nose (E); Permutation Plot of GX vs. YN E-nose (F).

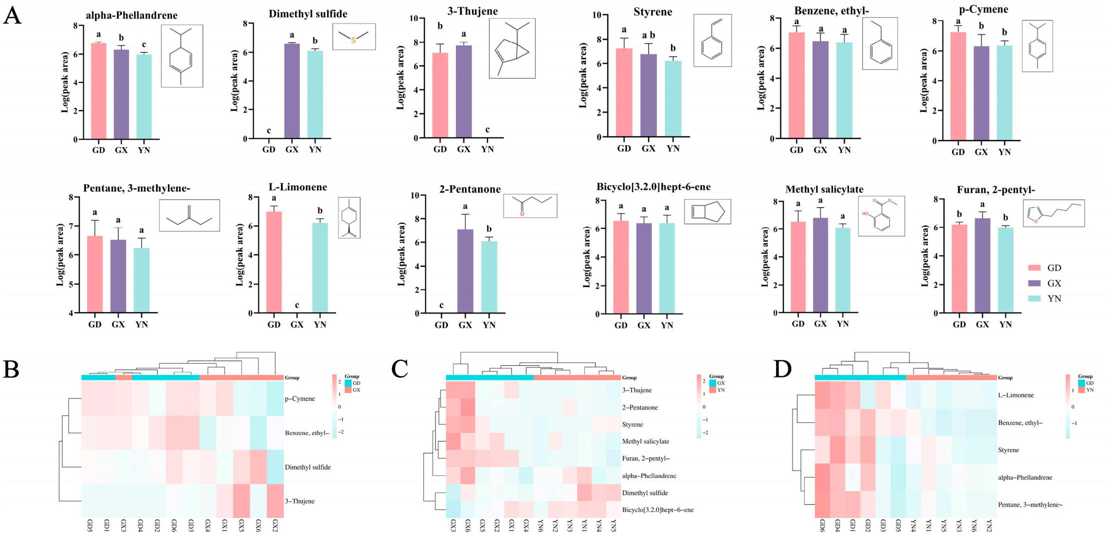

Fig S2: Bar chart of the content of 12 key differential compounds (A); Hierarchical clustering heatmap of differential compounds between GD and GX samples (B); Hierarchical clustering heatmap of differential compounds between GX and YN samples (C); Hierarchical clustering heatmap of differential compounds between GD and YN samples (D).
